# Diagnostic Performance of Serum Neutrophil–Lymphocyte and Serum Monocyte–Lymphocyte Ratios in Periprosthetic Joint Infection: A Comparative Meta-Analytic Review of 29 Studies

**DOI:** 10.3390/jcm14217645

**Published:** 2025-10-28

**Authors:** Rares-Mircea Birlutiu, Maryam Salimi, Serban Dragosloveanu, Cristian Scheau, Andreea Elena Vorovenci, Andrei Larie, Edoardo-Cristian Anea, Bogdan Neamtu, Victoria Birlutiu

**Affiliations:** 1 Department 14-Orthopedics, Anaesthesia Intensive Care Unit, Faculty of Medicine, “Carol Davila” University of Medicine and Pharmacy, 020021 Bucharest, Romania; 2 Department of Orthopaedics, “Foisor” Clinical Hospital of Orthopaedics, Traumatology and Osteoarticular TB, 021382 Bucharest, Romania; 3 McGovern Medical School, University of Texas Health Science Center, 6431 Fannin St., Houston, TX 77030, USA; 4 Department of Physiology, “Carol Davila” University of Medicine and Pharmacy, 050474 Bucharest, Romania; 5 Department of Radiology and Medical Imaging, “Foisor” Clinical Hospital of Orthopaedics, Traumatology and Osteoarticular TB, 021382 Bucharest, Romania; 6 Economic Cybernetics and Statistics Doctoral School, Bucharest University of Economic Studies, Piata Romana 6, 010371 Bucharest, Romania; 7 Faculty of Medicine, Lucian Blaga University of Sibiu, 550169 Sibiu, Romania; bogdan.neamtu@ulbsibiu.ro (B.N.);; 8 Pediatric Clinical Hospital Sibiu, 550166 Sibiu, Romania; 9 County Clinical Emergency Hospital, 550245 Sibiu, Romania

**Keywords:** periprosthetic joint infection, arthroplasty, neutrophil-to-lymphocyte ratio, monocyte-to-lymphocyte ratio, biomarkers, diagnostic accuracy, meta-analysis

## Abstract

**Background/Objectives:** Periprosthetic joint infection (PJI) remains one of the most devastating complications of arthroplasty, with early diagnosis crucial for successful management. The serum neutrophil–lymphocyte ratio (NLR) and monocyte–lymphocyte ratio (MLR) have been proposed as simple, inexpensive inflammatory biomarkers, but their diagnostic performance in PJI remains unclear. This meta-analysis aimed to compare the diagnostic accuracy of serum NLR and MLR in detecting PJI. Materials and **Methods:** A systematic literature search was conducted in PubMed, Web of Science, and Scopus up to April 2025. Twenty-nine eligible studies (*n* = 14,040 patients; 3418 with PJI, 10,622 without PJI) reporting diagnostic metrics for serum NLR or MLR were included. Extracted data comprised mean biomarker values, cut-off thresholds, sensitivity, specificity, and area under the receiver operating characteristic curve (AUC). Non-parametric statistical tests and subgroup analyses were applied to examine performance across infection types and PJI definitions. **Results:** Both serum NLR and MLR were significantly elevated in PJI patients compared with aseptic cases (*p* < 0.001 and *p* = 0.003, respectively). Pooled diagnostic accuracy was moderate: mean AUC 0.719 for NLR and 0.700 for MLR. For NLR, mean sensitivity was 69.9% and specificity 69.8%, with an average cut-off of 2.88. For MLR, mean sensitivity was 68.2% and specificity 70.4%, with an average cut-off of 0.34. Subgroup analyses indicated superior diagnostic performance of NLR in acute infections and variability depending on the PJI definition employed (*p* = 0.037). Strong correlations were observed between standardized mean differences in biomarker levels and corresponding diagnostic accuracy, particularly for NLR (ρ = 0.802, *p* = 0.002). **Conclusions:** Serum NLR demonstrates slightly superior diagnostic accuracy over serum MLR in identifying PJI, especially in acute settings. Both markers are inexpensive and widely accessible but show only moderate discriminative capacity, supporting their role as adjunctive rather than standalone diagnostic tools. Further large-scale prospective studies with harmonized methodologies are needed to refine biomarker thresholds and integrate them into multimodal diagnostic algorithms.

## 1. Introduction

Periprosthetic joint infection (PJI) is among the most severe and costly complications following total joint arthroplasty (TJA), including total knee and hip replacements [1,2,3,4,5]. Although its incidence remains relatively low, typically ranging between 0.5% and 2.5% in primary procedures [6,7,8], data from large registries and recent cohort studies estimate the incidence of periprosthetic joint infection (PJI) at approximately 0.5–2.3% following total hip arthroplasty (THA) [9] and around 1% after total knee arthroplasty (TKA). In shoulder arthroplasty, PJI rates range from ~1% to 4%, whereas elbow arthroplasty infections occur in 1.5–12.5% of cases [10]. Despite advances in perioperative prophylaxis and surgical techniques, recurrence and reinfection rates remain clinically significant. Following revision surgery, PJI recurs in an estimated 5–15% of cases, with reinfection rates consistently higher in knees than in hips [10]. PJI carries high morbidity, increases healthcare costs, and often necessitates complex revision surgeries [11]. With the global rise in joint replacements, the burden of PJI is expected to grow, amplifying the need for early, accurate, and accessible diagnostic tools [1,2,12]

Timely diagnosis of PJI remains a major clinical challenge. Current consensus criteria, including those from the Musculoskeletal Infection Society (MSIS), the International Consensus Meeting (ICM), and the European Bone and Joint Infection Society (EBJIS), combine clinical findings, serum and synovial biomarkers, microbiological cultures, and histopathologic data [13,14]. However, these protocols often rely on invasive tests, expensive synovial fluid analyses, or advanced laboratory resources that may not be available in all settings [15]. Moreover, no single test offers perfect sensitivity or specificity, particularly for low-grade or early infections where clinical symptoms may be muted and laboratory abnormalities subtle [16].

The serum neutrophil-to-lymphocyte ratio (NLR) and serum monocyte-to-lymphocyte ratio (MLR) have emerged as promising hematological biomarkers for distinguishing bacterial from viral infections and for predicting clinical outcomes across a range of conditions [17]. Among these, the serum NLR has attracted particular interest as a marker of systemic inflammation due to its established prognostic relevance. Biologically, it reflects the balance between neutrophils primary mediators of innate immunity that promote pro-inflammatory cascades and lymphocytes, which modulate adaptive immune responses. An elevated serum NLR indicates a shift toward a pro-inflammatory state [17,18,19].

The serum NLR has been investigated as a prognostic indicator in malignancy, sepsis, COVID-19, cardiovascular disease, diabetes, and, more recently, in periprosthetic joint infection (PJI) [18,19]. Inflammatory processes are commonly associated with elevated monocyte counts and reduced lymphocyte counts. The serum monocyte-to-lymphocyte ratio (MLR), may more accurately reflect the systemic inflammatory state than and have a prognostic value that has been demonstrated in diverse clinical settings, including cardiovascular disease, pneumonia, and chronic kidney disease [20]. Both serum NLR and serum MLR are inexpensive, rapidly obtainable from standard white blood cell differentials, and easily integrated into routine clinical workflows [21]. Nevertheless, evidence specifically addressing their diagnostic performance in PJI remains limited [22,23]. In a study of septic arthritis patients undergoing total hip arthroplasty, Jiang et al. reported that neither serum NLR nor serum MLR reliably detected occult infection [24]. By contrast, Varady et al. found that both serum NLR and synovial fluid NLR (SF-NLR) outperformed current clinical standards in diagnosing and prognosticating septic arthritis [25]. Similarly, Zhao et al. demonstrated elevated serum NLR and serum MLR values in patients with early PJI, suggesting potential utility for early postoperative infection detection [11].

Given these disparities a comprehensive synthesis of the available evidence is needed. This meta-analysis aims to systematically evaluate the diagnostic performance of conventional and emerging blood-based biomarkers for PJI. By pooling data on sensitivity, specificity, and AUC across multiple studies, we seek to clarify the role of these tools in clinical practice and provide evidence-based guidance for improving diagnostic accuracy in PJIs.

## 2. Materials and Methods

### 2.1. Study Design and Data Collection

This meta-analysis was designed to evaluate the diagnostic performance of the serum neutrophil-to-lymphocyte ratio (NLR) and serum monocyte-to-lymphocyte ratio (MLR) in distinguishing periprosthetic joint infection (PJI) from aseptic failure. Eligible studies were identified through a structured literature search and manually curated dataset, extracting summary-level data on diagnostic accuracy metrics, including mean values of serum NLR and serum MLR for PJI and non-PJI groups, area under the ROC curve (AUC), sensitivity, specificity, and diagnostic cut-off thresholds. Studies published between inception and 1 April 2025 were considered, and inclusion was restricted to those reporting original diagnostic data on PJI patients, with clearly defined infection criteria.

### 2.2. Data Extraction and Coding

For each study, we recorded year of publication, country of origin, sample size, number of PJI and non-PJI cases, and the diagnostic definition used for PJI. Diagnostic definitions were categorized as MSIS, ICM, EBJIS, IDSA, or combinations thereof. Infection phenotype was coded as acute, chronic, or mixed (acute + chronic). The primary variables extracted were:

- Serum NLR and serum MLR mean values for both PJI and non-PJI groups;
- Area under the ROC curve (AUC) for serum NLR and serum MLR;
- Sensitivity, specificity, and cut-off thresholds for each biomarker.

Each variable was extracted only if explicitly reported in the original study. No estimations or transformations were applied to derive missing values. A complete-case (listwise deletion) approach was used for all analyses.

### 2.3. Data Availability and Missing Data Management

Due to partial reporting in source articles, not all studies contributed data to each analysis. No imputation was performed. All analyses were restricted to studies with complete information on the variables involved.

### 2.4. Statistical Analysis

All statistical analyses were conducted using IBM SPSS Statistics (v.29). The statistical approach was structured as follows.

#### 2.4.1. Descriptive Statistics

Descriptive analyses were performed on diagnostic performance metrics (AUC, sensitivity, specificity, cut-off values) for both serum NLR and serum MLR. Measures of central tendency (mean, median) and dispersion (standard deviation, interquartile range) were reported. Data distributions were assessed via Shapiro–Wilk test.

#### 2.4.2. Between-Group Comparisons

Paired samples Wilcoxon signed-rank tests were conducted to compare serum NLR and serum MLR levels between PJI and non-PJI groups across studies that reported both values. Group comparisons of AUC values across infection definitions and infection types were conducted using the Kruskal–Wallis H test, due to non-normal distributions and unequal group sizes.

#### 2.4.3. Correlation Analyses

Spearman’s rank-order correlation was used to examine associations between diagnostic performance metrics (e.g., AUC, sensitivity, specificity, cut-off values), and between diagnostic accuracy (AUC) and both raw and standardized differences (SMD) in biomarker values between PJI and non-PJI groups.

The standardized mean difference (SMD) was calculated using the sample-size-weighted pooled SD $\left(S_{p}\right)$ (Equation (1)):

(1) $$\begin{array}{c} d=\frac{\left({\bar{X}}_{P.\mathrm{JI}\;}-{\bar{X}}_{\mathrm{nonP}.\mathrm{JI}}\right)}{S_{p}},\;\mathrm{with}\\ S_{p}=\sqrt{\left\{\left(n_{PJI}-1\right)SD_{PJI}^{2}+\left(n_{\mathrm{nonPJI}\;}-1\right)SD_{\mathrm{nonPJI}\;}^{2}\right\}/\left(n_{PJI}+n_{\mathrm{nonPJI}\;}-2\right)}\\ {\bar{X}}_{\mathrm{PJI}}=mean\;serum\;NLR\;or\;serum\;MLR\;value\;for\;PJI\;cases\\ {\bar{X}}_{\mathrm{nonPJI}}=mean\;serum\;NLR\;or\;serum\;MLR\;value\;for\;non-PJI\;cases\\ SD_{\mathrm{PJI}}=standard\;deviation\;for\;PJI\;group\\ SD_{\mathrm{nonPJI}}=standard\;deviation\;for\;non-PJI\;group\\ n_{\mathrm{PJI}},n_{\mathrm{nonPJI}\;}=\mathrm{group}\;\mathrm{sample}\;\mathrm{sizes};\\ S_{p}=\;\mathrm{pooled}\;\mathrm{SD}\;\mathrm{weighted}\;\mathrm{by}\;\mathrm{sample}\;\mathrm{sizes}.\\ \mathrm{Positive}\;\mathrm{SMD}\;\mathrm{indicates}\;\mathrm{higher}\;\mathrm{biomarker}\;\mathrm{values}\;\mathrm{in}\;\mathrm{the}\;\mathrm{PJI}\;\mathrm{group}. \end{array}$$

Equation (1)—SMD formula.

This allowed for a standardized measure of diagnostic separation across heterogeneous studies.

#### 2.4.4. Subgroup Analyses

Subgroup analyses were performed to investigate whether diagnostic accuracy differed by infection definition (MSIS, ICM, EBJIS, etc.) or by infection type (acute, chronic, mixed). AUC values for NLR and MLR were compared across subgroups using the Kruskal–Wallis test with post hoc inspection of mean ranks.

#### 2.4.5. Visualization

Boxplots were generated using IBM SPSS Statistics (v.29) to illustrate the distribution of AUC, sensitivity, specificity, and cut-off values across studies for both serum NLR and serum MLR. Visual inspection of these distributions provided further context for interpreting central tendencies, dispersion, and potential outliers.

### 2.5. HSROC Analyses

We also employed a hierarchical summary receiver operating characteristic (HSROC) approach, fitting a bivariate random-effects logistic-normal model (Reitsma) to each biomarker. The analysis was performed in R (v. 4.5.1, R Core Team, 2025). For each study, we reconstructed the 2 × 2 contingency table (true positives, false positives, false negatives, true negatives) from the reported sensitivity and specificity, along with the denominators. When necessary, we applied a continuity correction of 0.5. From the fitted model, we derived the summary sensitivity and specificity (on a probability scale), along with their 95% confidence intervals (using fixed-effects covariance). Additionally, we generated the HSROC curve and 95% prediction regions (using between-study covariance). The area under the curve (AUC) was calculated from the HSROC on a trimmed false positive rate grid to avoid boundary instabilities.

### 2.6. Ethical Considerations

This study is based exclusively on previously published data and did not involve human subjects directly. Ethical approval was not required.

### 2.7. PRISMA Flow

**Figure 1:**
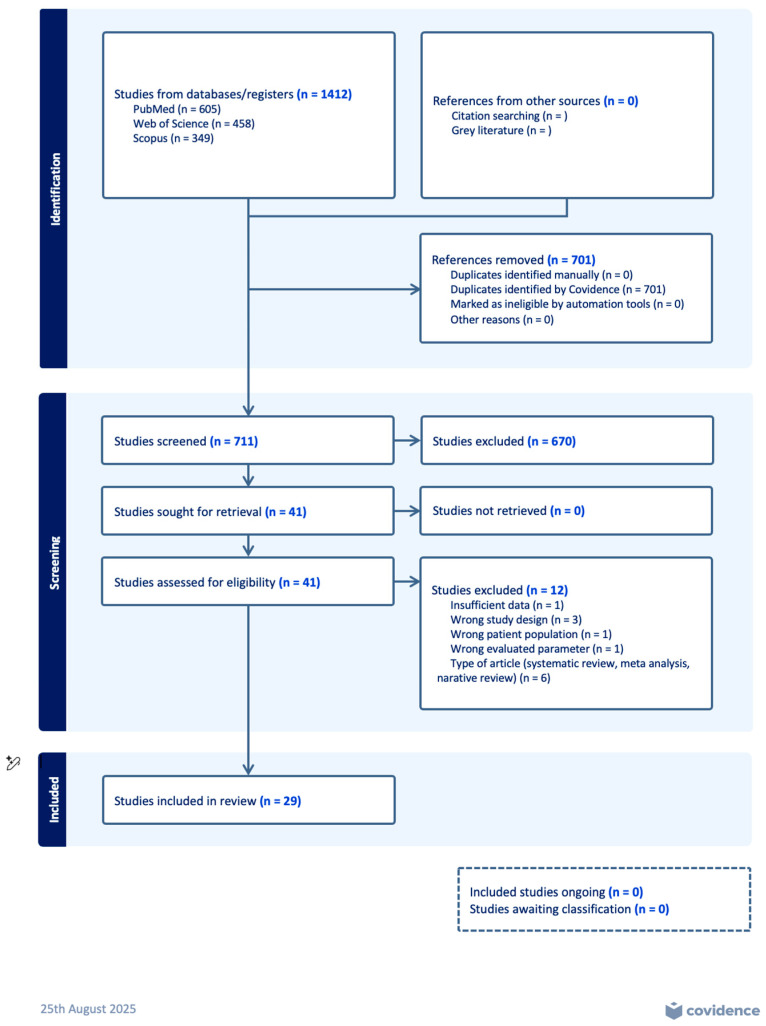
Flow diagram of included studies in the article.

This meta-analysis followed the PRISMA 2020 (Preferred Reporting Items for Systematic Reviews and Meta-Analyses) [11] guidelines for study identification, screening, eligibility, and inclusion (see Figure 1). A structured search was conducted in PubMed, Web of Science, and Scopus databases, using a combination of MeSH terms and free-text keywords related to *periprosthetic joint infection*, *serum neutrophil-to-lymphocyte ratio (NLR)*, and *serum monocyte-to-lymphocyte ratio (MLR)*. The search strategy was not restricted by language but was limited to studies published between inception, and 1 April 2025. After removal of duplicates, two independent reviewers screened titles and abstracts for eligibility. Full-text screening was conducted for studies reporting diagnostic performance metrics (AUC, sensitivity, specificity, and cut-off values) or biomarker levels in both PJI and non-PJI groups. Disagreements were resolved by consensus. Studies not providing quantitative data or using non-standard definitions of PJI were excluded. The completed PRISMA checklist is provided in Supplementary Material.

## 3. Results

Our analysis was based on summary statistics extracted from 29 eligible studies published between 2016 and 2025 [3,4,11,15,16,24,26,27,28,29,30,31,32,33,34,35,36,37,38,39,40,41,42,43,44,45,46,47]. Most studies were published in 2023 (*n* = 10), followed by 2022 (*n* = 6), 2021 (*n* = 5), 2024 (*n* = 3), 2020 and 2025 (*n* = 2 each), and 2016 (*n* = 1), reflecting a recent increase in publications addressing diagnostic biomarkers in periprosthetic joint infection. The included studies were conducted in eight countries, with the largest contributions from China (*n* = 13) and the United States (*n* = 6), followed by Germany (*n* = 3), Canada and Turkey (*n* = 2 each), and Austria, Italy, and Japan (*n* = 1 each). The total pooled sample size was 14,040 patients, comprising 3418 patients with PJI and 10,622 without PJI. Regarding PJI case definitions, 12 studies employed the MSIS criteria, 9 used the ICM definition, 3 combined MSIS and EBJIS, 2 applied the IDSA definition, 2 used EBJIS alone, and 1 study used MSIS + IDSA. In terms of infection chronicity, 14 studies included both acute and chronic PJI cases, 9 focused solely on chronic infections, 5 addressed acute infections, and 1 study did not specify the infection type.

Due to partial reporting in the source articles, some variables presented missing values. Specifically, 26 studies reported the mean serum NLR values for PJI patients [3,4,11,16,24,27,28,29,30,31,32,33,34,35,36,37,38,39,40,41,43,44,45,46,47], and 25 studies for non-PJI patients [3,4,11,16,24,27,28,29,30,31,32,33,34,35,36,38,39,40,41,43,44,45,46,47]. The area under the curve (AUC) was reported in 27 studies [3,4,11,15,16,24,26,27,28,29,30,31,32,33,34,35,36,37,38,39,40,41,42,43,44,45,46,47], while sensitivity and specificity of serum NLR were available in 28 studies each [3,4,11,15,16,24,26,27,28,29,30,32,33,34,35,36,37,38,39,40,41,42,43,44,45,46,47]. Cut-off thresholds for serum NLR were reported in all studies. Regarding serum MLR, 11 studies reported the mean values for both PJI and non-PJI patients [16,24,27,28,29,30,31,40,41,43,44]. The serum MLR AUC was reported in 8 studies [16,24,28,29,40,41,43,44], while sensitivity and specificity of MLR were available in 10 studies each [16,24,27,28,29,30,40,41,43,44]. Cut-off thresholds for serum MLR were reported in 11 studies [16,24,27,28,29,30,31,40,41,43,44]. All statistical analyses were conducted using complete-case analysis (listwise deletion), and no data imputation was performed. Each analysis included only those studies with available data for the variables involved. The availability of data analyzed in this study is reported in Table 1.

### 3.1. Comparison of Serum NLR and Serum MLR Between PJI and Non-PJI Groups

To assess whether the serum neutrophil-to-lymphocyte ratio and serum monocytes-to-lymphocyte ratio differs between PJI and non-PJI patients, we conducted a paired samples *t*-test on the mean serum NLR and serum MLR values reported per study. Only studies reporting both PJI and non-PJI means were included (*n* = 25 for serum NLR and *n* = 11 for serum MLR). Missing data were handled using listwise deletion, and no imputation was applied. The Shapiro–Wilk test indicated that the differences in serum NLR and serum MLR between PJI and non-PJI groups were not normally distributed (W = 0.785, *p* < 0.001 for NLR, and W = 0.743, *p* < 0.001 for MLR). Therefore, a non-parametric Wilcoxon signed-rank test was used for the paired comparison. A Wilcoxon signed-rank test was conducted to evaluate the difference in serum NLR between PJI and non-PJI cases across 25 studies, and serum MLR between PJI and non-PJI cases across 11 studies. All studies reported higher serum NLR values for the PJI group (25 negative ranks, 0 positive ranks, 0 ties), and also higher serum MLR values for the PJI group (11 negative ranks, 0 positive ranks, 0 ties) indicating a consistent trend. The test showed a statistically significant difference (Z = −4.372 with a *p* < 0.001 for serum NLR, and Z = −2.940 with a *p* = 0.003 for serum MLR), suggesting that both serum NLR and serum MLR are substantially elevated in patients with PJI compared to those without.

### 3.2. Assessment of the Diagnostic Performance of Serum NLR and Serum MLR Across Studies

Descriptive statistics were calculated for the diagnostic performance of serum NLR and serum MLR across studies, including the area under the ROC curve (AUC), sensitivity, specificity, and cut-off values. Analyses were conducted on available data using complete-case analysis, without imputation. The variables were treated as continuous, and measures of central tendency and dispersion were reported. A total of 29 studies reported serum NLR diagnostic thresholds, with 27 reporting the area under the ROC curve (AUC), and 28 reporting sensitivity and specificity. A total of 11 studies reported serum MLR diagnostic thresholds, with 8 reporting the area under the ROC curve (AUC), and 10 reporting sensitivity and specificity. Descriptive analysis was conducted using only complete cases for each indicator, with no imputation of missing values.

Descriptive statistics were computed for the diagnostic performance of the serum neutrophil-to-lymphocyte ratio (serum NLR) across 26 studies with complete data. The mean area under the curve (AUC) for serum NLR was 0.719 (SD = 0.082), indicating moderate diagnostic accuracy. Sensitivity values ranged from 46.7% to 94.7%, with a mean of 69.86% (SD = 12.10), while specificity ranged from 38.2% to 89.7%, with a mean of 69.84% (SD = 12.39). The reported diagnostic cut-off values for serum NLR varied between 2.10 and 4.77, with a mean threshold of 2.88 (SD = 0.69). These results suggest substantial variability in reported diagnostic performance across studies, though the average sensitivity and specificity were balanced around 70%. Descriptive statistics were computed also for the diagnostic performance of the serum monocytes-to-lymphocyte ratio (serum MLR) across 8 studies with complete data. The mean area under the curve (AUC) for serum MLR was 0.700 (SD = 0.080), indicating moderate diagnostic accuracy. Sensitivity values ranged from 54.10% to 82.9%, with a mean of 68.23% (SD = 10.61), while specificity ranged from 37.5% to 83.6%, with a mean of 70.36% (SD = 16.04). The reported diagnostic cut-off values for serum MLR varied between 0.22 and 0.45, with a mean threshold of 0.34 (SD = 0.10). These results also suggest for serum MLR an substantial variability in reported diagnostic performance across studies, though the average sensitivity and specificity were again balanced around 70%.

The boxplot of AUC values for serum NLR reported across studies shows a median diagnostic accuracy around 0.72, with most values falling between 0.65 and 0.80. One study reported a substantially lower AUC (~0.51), which was identified as a potential outlier. Despite this, the overall distribution of AUC for serum NLR values was moderately symmetric, with no extreme skewness observed. These findings support the use of serum NLR as a moderately accurate diagnostic biomarker for PJI across heterogeneous study populations. The boxplot of AUC values for serum MLR reported across studies shows a median diagnostic accuracy around 0.72, with most values falling between 0.63 and 0.76. The overall distribution of AUC for serum MLR values was moderately symmetric, with no extreme skewness observed. These findings support also the use of serum MLR as a moderately accurate diagnostic biomarker for PJI across heterogeneous study populations. The boxplots for AUC values are highlighted in Figure 1.

The distribution of serum NLR sensitivity across studies showed a relatively balanced spread, with a median sensitivity around 71%. Most values fell within an interquartile range of approximately 63% to 82%, indicating moderate consistency across studies. The minimum reported sensitivity was about 47%, while the maximum reached nearly 95%, reflecting substantial heterogeneity in diagnostic performance depending on study design or population. No extreme outliers were observed, suggesting a stable pattern in reported sensitivity estimates for serum NLR as a diagnostic biomarker. The distribution of serum MLR sensitivity across studies showed also a relatively balanced spread, with a median sensitivity around 66%. Most values fell within an interquartile range of approximately 59% to 77%, indicating a similar to serum NLR moderate consistency across studies. The minimum reported sensitivity was about 54.10%, while the maximum reached nearly 82.80%, reflecting substantial heterogeneity in diagnostic performance depending on study design or population. No extreme outliers were observed also for this serum parameter, suggesting again a stable pattern in reported sensitivity estimates for serum MLR as a diagnostic biomarker. The boxplots for sensitivity for both serum parameters are highlighted in Figure 1.

**Figure 2:**
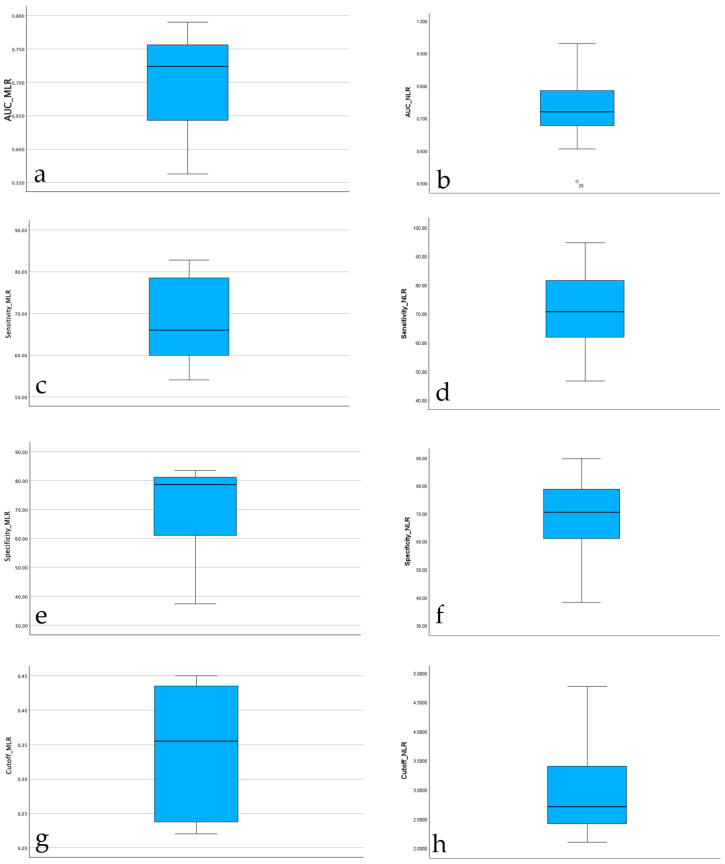
Boxplots for serum NLR and MLR. (**a**) Boxplot of AUC values for serum MLR; (**b**) Boxplot of AUC values for serum NLR; (**c**) Boxplot of sensitivity for serum MLR; (**d**) Boxplot of sensitivity for serum NLR; (**e**) Boxplot of specificity for serum MLR; (**f**) Boxplot of specificity for serum NLR; (**g**) Boxplot of cut-offs for serum MLR; (**h**) Boxplot of cut-offs for serum NLR.

The boxplot of serum NLR specificity values indicates a median specificity around 70%, closely mirroring the distribution observed for sensitivity. The interquartile range was approximately 62% to 78%, suggesting moderate consistency across studies. The lowest reported specificity was ~38%, while the highest reached ~90%, again highlighting heterogeneity in diagnostic performance. Despite this variation, the overall spread appears symmetric, with no extreme outliers, confirming the robustness of most reported values. The boxplot of serum MLR specificity values indicates a median specificity around 79%, better then the distribution observed for sensitivity. The interquartile range was approximately 57% to 84%, suggesting moderate consistency across studies. The lowest reported specificity was ~37%, while the highest reached ~84%, again highlighting heterogeneity in diagnostic performance. Despite this variation, the overall spread appears symmetric, with no extreme outliers, confirming the robustness of most reported values. The boxplots for specificity for both serum evaluated parameters are highlighted in Figure 2.

The distribution of diagnostic cut-off values for serum NLR across studies shows a median of approximately 2.7, with most values falling between 2.2 and 3.5. The minimum observed value was 2.1, and the maximum reached 4.77, indicating moderate variability in the threshold values used for diagnosing PJI. The shape of the distribution is slightly right-skewed, but no extreme outliers were identified. This suggests that while most studies used similar thresholds (around 2.5–3), some reported higher cut-offs, which may reflect differences in methodology, population characteristics, or the definition of infection used. The distribution of diagnostic cut-off values for serum MLR across assessed studies shows a median of approximately 0.35, with most values falling between 0.25 and 0.42. The minimum observed value was 0.22, and the maximum reached 0.45, indicating moderate variability in the threshold values used for diagnosing PJI. The shape of the distribution is center-skewed. This suggests that while most studies used similar thresholds, regardless the fact that some reported slightly higher cut-offs. The boxplots for cut-offs for both serum evaluated parameters are highlighted in Figure 1.

### 3.3. Assessment of Associations Between Diagnostic Performance Metrics of Serum NLR

To explore potential associations between diagnostic performance metrics, we computed Spearman’s rank correlation coefficients between AUC_NLR, sensitivity, specificity, and the serum NLR cutoff values. This non-parametric test was chosen due to the lack of normal distribution, as confirmed by Shapiro–Wilk test and visual inspections (boxplots). Listwise deletion was applied for missing values.

Our results indicated several statistically significant correlations.

There was a moderate positive correlation between AUC_NLR and Sensitivity_NLR (ρ = 0.562, *p* = 0.003), suggesting that studies reporting higher AUCs tend to also report higher sensitivity. AUC_NLR was also positively correlated with Specificity_NLR (ρ = 0.461, *p* = 0.018), indicating a simultaneous increase in specificity alongside AUC. Specificity_NLR and Cutoff_NLR were weakly positively correlated (ρ = 0.399, *p* = 0.043), suggesting that studies with higher specificity tend to report slightly higher cutoff thresholds. No statistically significant correlations were observed between Cutoff_NLR and AUC_NLR (*p* = 0.384), nor between Sensitivity_NLR and Cutoff_NLR (*p* = 0.285).

These results reflect a general trend in which both sensitivity and specificity appear to increase in tandem with AUC values, while the choice of serum NLR cutoff remains variable and less consistently associated with diagnostic performance metrics. A summary of Spearman Correlations Between Diagnostic Metrics is reported in Table 2.

### 3.4. Assessment of Associations Between Diagnostic Performance Metrics of Serum MLR

To explore potential associations between diagnostic performance metrics, we computed Spearman’s rank correlation coefficients between AUC_MLR, sensitivity, specificity, and the serum MLR cutoff values. This non-parametric test was chosen due to the lack of normal distribution, as confirmed by Shapiro–Wilk test and visual inspections (boxplots). Listwise deletion was applied for missing values.

Our results indicated no statistically significant correlations. A summary of Spearman Correlations Between Diagnostic Metrics is reported in Table 3. While none of the pairwise associations achieved statistical significance at the conventional *p* = 0.05 threshold, the directional trends aligned with clinical expectations. Both sensitivity and specificity tended to increase proportionally with AUC_MLR, albeit at different rates. Conversely, the diagnostic threshold for MLR while moderately correlated with specificity (ρ = 0.168) and AUC (ρ = 0.611) did not exhibit a consistent or statistically meaningful relationship with overall test accuracy. While the upward trends in sensitivity and specificity reinforce AUC performance, merely adjusting the serum MLR threshold appears insufficient to achieve significant diagnostic gains. These findings underscore the importance of integrated biomarker interpretation over isolated cut-off optimization.

### 3.5. Assessment of Whether the Diagnostic Accuracy (AUC) of Serum NLR Differs Depending on the PJI Definition Used

**Figure 3:**
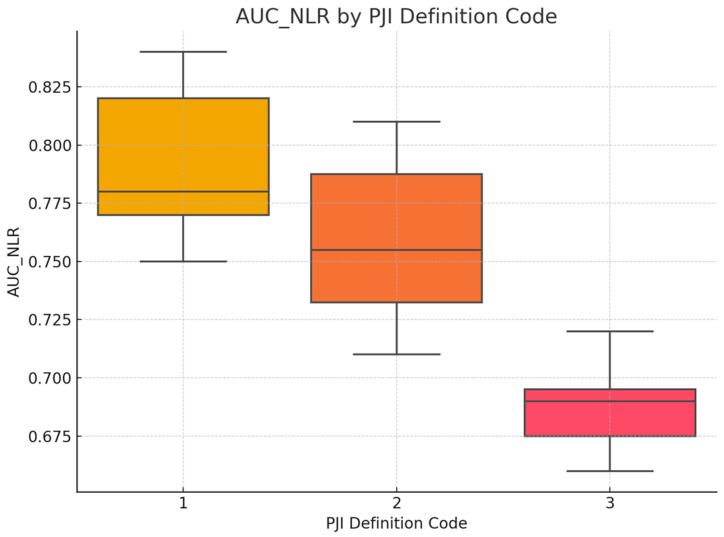
AUC_NLR by PJI Definition Code. The comparative boxplot illustrates the distribution of AUC_NLR values stratified by the periprosthetic joint infection (PJI) definition employed: Group 1 = MSIS criteria, Group 2 = ICM criteria, and Group 3 = alternative or non-standard definitions. Group 1 demonstrated the highest average AUC_NLR with tighter interquartile dispersion, suggesting both superior and more consistent diagnostic performance when standardized criteria are applied. Group 2 displayed an intermediate mean AUC but with greater variability, likely reflecting the broader spectrum of cases captured under the ICM framework. In contrast, Group 3 studies—those utilizing institution-specific or less-defined criteria—exhibited the lowest AUC values and minimal variability, potentially reflecting both limited diagnostic robustness and a narrower application of the biomarker under ambiguous case definitions.

To assess whether the diagnostic accuracy of serum NLR (measured by AUC) differed according to the PJI definition used, a between-group comparison was performed. Initially, the distribution of AUC_NLR was tested for normality within each group defined by *PJI_Definition_Code* using both the Kolmogorov–Smirnov and Shapiro–Wilk tests. While the MSIS and ICM groups showed no significant deviations from normality, the other groups were either too small for valid testing or showed signs of non-normality. Given the small and unequal group sizes, as well as violations of normality assumptions in several groups, the non-parametric Kruskal–Wallis H test was selected as the appropriate method for comparing AUC values across PJI definition categories. The AUC_NLR was entered as the dependent variable, and *PJI_Definition_Code* served as the grouping variable (coded 1 to 6). Statistical significance was set at *p* < 0.05. The Kruskal–Wallis H test revealed a statistically significant difference in serum NLR diagnostic accuracy (AUC values) across the PJI definition groups, H(5) = 11.87, *p* = 0.037. This suggests that the diagnostic performance of serum NLR varies depending on the definition of periprosthetic joint infection applied. Specifically, the highest mean rank was observed in the *MSIS + EBJIS* group (Mean Rank = 24.50), followed by the *IDSA* group (21.25), while the *EBJIS* group showed the lowest mean rank (4.50), indicating a potential variation in biomarker performance under different diagnostic frameworks, see Figure 3. However, given the limited sample sizes in some groups (e.g., *n* = 1–3), these differences should be interpreted cautiously.

### 3.6. Assessment of Whether the Diagnostic Accuracy (AUC) of Serum MLR Differs Depending on the PJI Definition Used

**Figure 4:**
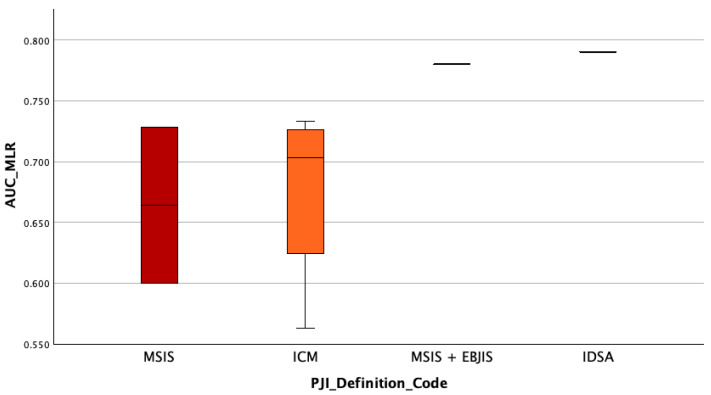
AUC_MLR by PJI Definition Code. Boxplot illustrating the distribution of AUC_MLR values across PJI diagnostic criteria: MSIS, ICM, MSIS + EBJIS, and IDSA. While no statistically significant differences were observed (*p* = 0.253), studies using MSIS + EBJIS and IDSA definitions showed higher AUCs, suggesting potential performance advantages with more structured or comprehensive criteria.

We also assessed whether the diagnostic accuracy of serum MLR (measured by AUC) is different according to the PJI definition used, a between-group comparison was performed also performed. The same steps that have been performed for AUC_MLR have also been performed in this case. The Kruskal–Wallis H test revealed no statistically significant difference in MLR diagnostic accuracy (AUC values) across the PJI definition groups, H(3) = 4.08, *p* = 0.253. This suggests that the diagnostic performance of serum MLR does not varies depending on the definition of periprosthetic joint infection applied. The highest mean rank was observed in the *IDSA* group (Mean Rank = 8.00), followed by the *MISI + EBJIS* group (7.00), while the *ICM and MSIS* groups showed the lowest mean ranks (3.50). However, given the limited sample sizes in some groups (e.g., *n* = 1–4), these differences should be interpreted cautiously, see Figure 4.

### 3.7. Assessment of Whether the Infection Type (Acute, Chronic, Mixed) Influences the Diagnostic Performance of Serum NLR (AUC Values)

To investigate whether the type of infection influenced the diagnostic accuracy of serum NLR, a Kruskal–Wallis H test was conducted. The variable *Infection_Type_Code* categorized studies as reporting acute, chronic, or mixed infections. Studies with unknown infection type were excluded from this analysis. Due to the unequal group sizes and the non-normal distribution of AUC values, the non-parametric Kruskal–Wallis test was deemed appropriate. Statistical significance was set at *p* < 0.05. A Kruskal–Wallis H test was performed to determine whether the diagnostic performance of serum NLR (as measured by AUC) varied based on the type of infection reported in the studies. The results indicated a statistically significant difference in AUC_NLR between infection types, H(2) = 8.64, *p* = 0.013.

Post hoc inspection of mean ranks revealed that:

- Studies with acute infections had the highest mean rank (22.63);
- Followed by those with chronic infections (14.81);
- While studies including both acute and chronic cases (*mixed type*) showed the lowest mean rank (10.14).

These findings suggest that the diagnostic performance of serum NLR may be influenced by the infection type, potentially showing higher discriminatory ability in studies focused solely on acute infections. The findings suggest that the diagnostic accuracy of serum NLR in detecting periprosthetic joint infection (PJI) is not uniform across infection types. Specifically, studies focusing exclusively on acute infections reported significantly higher AUC values compared to those including chronic or mixed (acute and chronic) infections.

This trend may reflect underlying differences in the host immune response and systemic inflammation. Acute infections are typically associated with more pronounced neutrophilia and lymphopenia, leading to elevated serum NLR values and potentially better discriminatory power. In contrast, chronic or low-grade infections may induce a more subtle systemic response, diminishing the ability of serum NLR to differentiate between septic and aseptic cases. These results highlight the importance of clinical context when interpreting biomarker performance. Pooling studies with heterogeneous infection types may underestimate the true diagnostic value of serum NLR in specific clinical scenarios. Therefore, stratifying future biomarker analyses by infection type may enhance diagnostic precision and guide tailored clinical decision-making.

### 3.8. Assessment of Whether the Infection Type (Acute, Chronic, Mixed) Influences the Diagnostic Performance of Serum MLR (AUC Values)

To assess whether the diagnostic performance of the serum monocyte-to-lymphocyte ratio (MLR) varies across infection phenotypes, we conducted a non-parametric Kruskal–Wallis H test comparing AUC_MLR values across three clinical categories: acute, chronic, and mixed (acute + chronic) periprosthetic joint infections (PJI) similar to the previous analysis. Post hoc inspection of mean ranks revealed the highest AUC_MLR values in acute infections (mean rank = 6.00), followed by chronic cases (5.00), with the lowest diagnostic performance observed in mixed infection cohorts (2.33). While underpowered, this rank gradient aligns with biological plausibility—acute infections often trigger a stronger systemic inflammatory response, enhancing the discriminatory value of hematologic biomarkers such as serum MLR. While not statistically conclusive, the observed ranking supports the hypothesis that serum MLR-based discrimination may perform better in acutely inflamed prosthetic joints, where systemic hematologic shifts are more pronounced. These findings underscore the need for stratified biomarker evaluation and larger datasets to robustly characterize performance across infection subtypes.

### 3.9. Association Between Serum NLR Differential and Diagnostic Accuracy (AUC_NLR)

To evaluate whether greater separation in serum neutrophil-to-lymphocyte ratio (NLR) values between infected and non-infected patients correlates with improved diagnostic performance, we conducted a non-parametric Spearman correlation analysis between the mean difference in serum NLR values (NLR_Diff = NLR_PJI_Mean − NLR_nonPJI_Mean) and the reported area under the ROC curve (AUC_NLR) across eligible studies. The analysis demonstrated a statistically significant positive correlation (ρ = 0.567, *p* = 0.005), indicating that studies reporting a wider divergence in serum NLR between septic and aseptic cases also tended to report higher diagnostic accuracy. These findings support the hypothesis that the magnitude of the intergroup serum NLR difference is an important determinant of the biomarker’s discriminatory strength. However, it is essential to interpret these results within context. AUC is a comprehensive measure of diagnostic performance that incorporates distributional overlap and threshold behavior, whereas NLR_Diff reflects only the absolute difference in group means, without accounting for within-group variability. Consequently, studies with identical NLR_Diff values may yield divergent AUCs depending on standard deviations and distributional skew. While this analysis does not substitute for a formal meta-analysis of effect sizes, it nonetheless reinforces the clinical relevance of the biological gradient underpinning serum NLR performance in PJI diagnostics.

### 3.10. Association Between Serum MLR Differential and Diagnostic Performance (AUC_MLR)

To investigate whether a greater difference in serum monocyte-to-lymphocyte ratio (MLR) between PJI and non-PJI cases is associated with enhanced diagnostic accuracy, we performed a non-parametric Spearman correlation between the absolute serum MLR differential (MLR_Diff = MLR_PJI_Mean − MLR_nonPJI_Mean) and the area under the ROC curve (AUC_MLR). The analysis yielded a moderate positive correlation (ρ = 0.548); however, this association did not reach statistical significance (*p* = 0.160), likely due to the limited number of observations (n = 8). Although underpowered, the observed trend suggests that a greater separation in serum MLR values may correspond with improved discriminative capacity. These findings are consistent with the conceptual model wherein systemic inflammatory markers, such as serum MLR, reflect underlying immune activation, particularly in acute infectious states. However, due to the small sample size and lack of statistical significance, this relationship remains hypothesis generating and warrants further validation in larger, methodologically harmonized datasets.

### 3.11. Relationship Between the Magnitude of the Standardized Difference in Serum NLR and Serum MLR Values (SMD) and the Diagnostic Accuracy (AUC) Across Studies

To quantify the magnitude of difference in serum NLR and serum MLR values between patients with and without PJI across studies, we computed the Standardized Mean Difference (SMD). This approach accounts for differences in measurement scales and variability between groups, providing a unitless effect size that facilitates comparison across heterogeneous samples.

The formula no. 1 represents Cohen’s d based on pooled variance and is appropriate when comparing two independent groups with potentially different sample sizes and variances. By standardizing the mean difference, the SMD provides a more interpretable and comparable estimate of effect size across studies than raw mean differences alone.

We selected this metric to complement the simple arithmetic difference (NLR_Diff or MLR_Diff), allowing for a more robust assessment of the relationship between the discriminatory power of the biomarker (AUC) and the magnitude of between-group separation. The use of both raw and standardized measures strengthens the reliability of our findings and enhances cross-study interpretability in meta-analytical contexts.

To explore the relationship between the magnitude of the standardized difference in serum NLR values (SMD) and the diagnostic accuracy (AUC) across studies, Spearman’s rank-order correlation was performed. The analysis revealed a strong and statistically significant positive association between the two indicators (ρ = 0.802, *p* = 0.002), suggesting that studies reporting larger standardized differences in serum NLR between PJI and non-PJI groups tend to also report higher diagnostic performance (AUC). This supports the notion that the discriminatory power of NLR increases with the magnitude of the difference between infected and non-infected patients. Also, a nonparametric Spearman’s rank correlation was conducted to assess the relationship between the AUC of MLR and the SMD in serum MLR across studies (N = 6). The analysis revealed a positive, moderate correlation between AUC_MLR and SMD_MLR (ρ = 0.657), though this did not reach statistical significance (*p* = 0.156). These findings suggest a trend toward an association between higher discriminatory power of serum MLR (as measured by AUC) and greater between-group separation (as quantified by SMD), albeit in a small sample size limiting the statistical power.

### 3.12. HSROC Analyses

**Figure 5:**
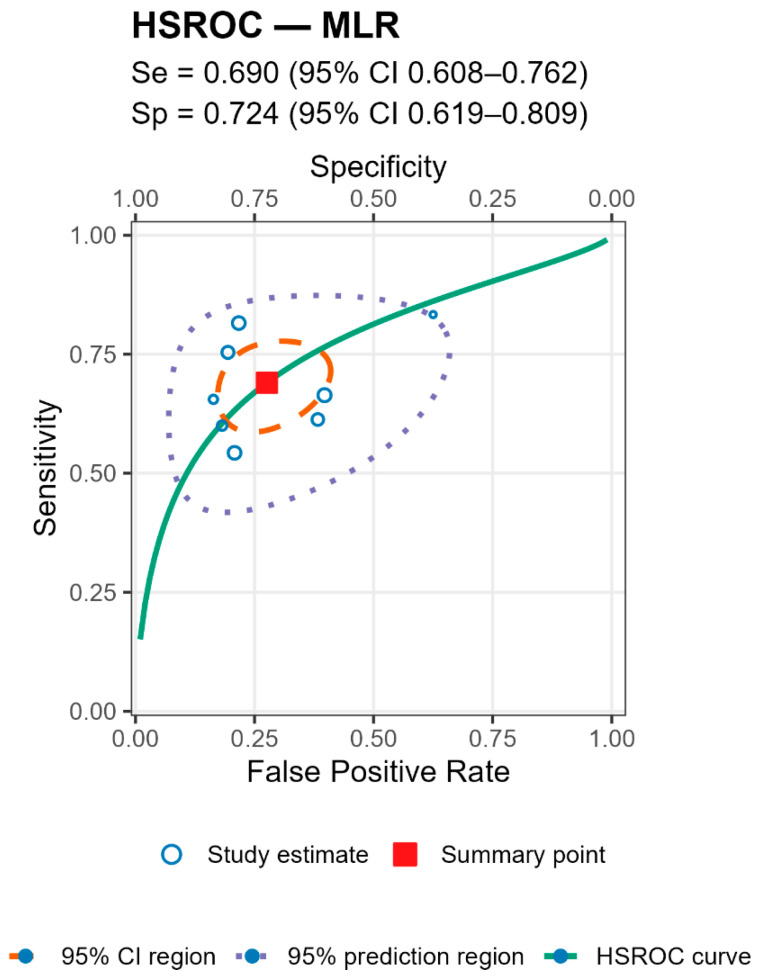
HSROC from the bivariate Reitsma model. Solid curve = summary ROC; filled square = pooled operating point (sensitivity and specificity); dashed ellipse = 95% confidence region; dotted ellipse = 95% prediction region; circles = individual study estimates (size ∝ sample size). Summary sensitivity was 0.690 (95% CI 0.608–0.762) and specificity 0.724 (95% CI 0.619–0.809), AUC ≈ 0.750.

**Figure 6:**
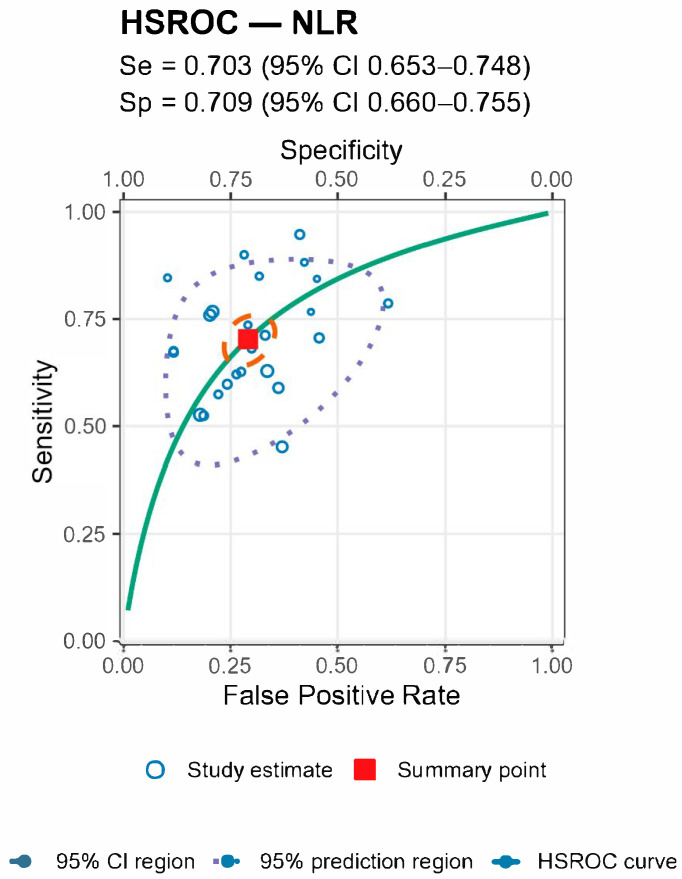
HSROC from the bivariate Reitsma model. Solid curve = summary ROC; filled square = pooled operating point (sensitivity and specificity); dashed ellipse = 95% confidence region; dotted ellipse = 95% prediction region; circles = individual study estimates (size ∝ sample size). The HSROC yielded a summary sensitivity of 0.703 (95% CI 0.653–0.748) and specificity of 0.709 (95% CI 0.660–0.755), with AUC ≈ 0.755.

For both markers the prediction regions were wider than the confidence regions, indicating substantial between-study heterogeneity (Figure 5 and Figure 6 and Table 4).

Stratified analyses by infection chronicity, PJI definition, are shown in Tables/Figures and suggest that performance varies with case-mix and case definition: NLR is more sensitive in acute infection (mixed acute+chronic cohorts dilute accuracy), and specificity is highest when PJI is defined by MSIS+EBJIS, lower under MSIS or ICM. For MLR, subgroup differences are small/non-significant, with a weak trend toward higher sensitivity in chronic cohorts. By infection chronicity, NLR showed higher pooled sensitivity in acute infection than in mixed acute+chronic cohorts (0.77 [95% CI 0.71–0.83] vs. 0.66 [0.61–0.72]; Wald z = 2.68, *p* = 0.007), with broadly similar specificity; AUCs were 0.78 (acute), 0.78 (chronic), and 0.71 (mixed). For MLR, subgroup differences by chronicity were not statistically significant, though sensitivity and AUC trended higher in chronic cohorts (Se 0.70 [0.56–0.82], AUC 0.77) than in mixed cohorts (Se 0.57 [0.52–0.62], AUC 0.56). By PJI definition (NLR), sensitivities were comparable, whereas specificity was highest with MSIS+EBJIS (0.85 [0.78–0.91]) and lower under MSIS (0.71 [0.63–0.79]) and ICM (0.65 [0.56–0.73]); Wald contrasts indicated higher false-positive rates for MSIS (z = 2.54, *p* = 0.011) and ICM (z = 3.53, *p* < 0.001) versus MSIS+EBJIS. Full subgroup HSROC plots and pooled estimates are shown in the Figures/Tables section (Appendix A).

### 3.13. Risk of Bias Assessment

The risk of bias of the included studies was assessed using the QUADAS-2 tool across the domains of patient selection, index test, reference standard, and flow and timing. Overall, most studies demonstrated a low risk of bias in the reference standard domain, as the majority applied established definitions for periprosthetic joint infection (MSIS, EBJIS, or ICM criteria). However, patient selection bias was frequently present, since nearly all studies were retrospective single-center cohorts, and several excluded patients without complete laboratory data or used non-consecutive enrolment strategies. The index test domain was rated as moderate to high risk in many studies, largely due to the derivation of NLR/MLR cutoffs post hoc within the same cohorts, raising the potential for overestimation of diagnostic accuracy. Flow and timing were often unclear or at moderate risk, as the interval between blood sampling and reference testing was not consistently reported, and some studies excluded patients during follow-up. Only a minority of studies achieved low risk across all domains. Collectively, these findings suggest that while the overall methodological quality was acceptable, the predominance of retrospective designs and exploratory thresholds should be considered when interpreting the pooled estimates; see Table 5.

## 4. Discussion

This meta-analysis synthesized evidence from 29 studies published between 2016 and 2025 that evaluated the diagnostic performance of the serum neutrophil-to-lymphocyte ratio (NLR) and serum monocyte-to-lymphocyte ratio (MLR) in periprosthetic joint infection (PJI). Across pooled analyses that included a sample size of 14,040 patients, comprising of 3418 patients with PJI and 10,622 without PJI, serum NLR consistently demonstrated higher diagnostic accuracy than serum MLR, with both markers exhibiting moderate discriminative capacity overall. The mean AUC for serum NLR was 0.719 with a mean sensitivity of 69.86% and specificity of 69.84% while the AUC for serum MLR was 0.700 with a mean sensitivity of 68.23% and specificity of 70.36%. Subgroup analyses revealed that diagnostic performance was influenced by the definition of PJI applied and by infection chronicity. Both serum NLR and MLR tended to perform better in acute PJIs compared with chronic or mixed reported cases. Furthermore, correlation analyses demonstrated a moderate positive relationship between the magnitude of biomarker differences (absolute and standardized mean differences) and the corresponding diagnostic accuracy as measured by area under the receiver operating characteristic curve.

Our findings align with an emerging body of literature highlighting the utility of serum NLR in musculoskeletal infections. Similar sensitivities and specificities have been reported also by Jiao J. Huang et al. and Wu H. et al. [4,47]. Better sensitivities but with lower specificities have been reported in studies that included a low number of patients both with PJI (n = ranged from 26 to 78) and non-PJI cases [3,11,27,42].

Although the serum neutrophils-to-lymphocytes ratio and serum monocyte-to-lymphocyte ratio showed promising results in diagnosing other infectious conditions such as appendicitis, surgical site infections or bloodstream infections but could only obtain moderate results when diagnosing PJI also in a meta-analysis [48,49,50]. With sensitivities of about 70% and specificities of about 70%, these parameters did not aid in diagnosing PJI, similar results and conclusion have also been reported by Sigmund I. et al. [27].

In agreement with the present analysis results, Varady et al. demonstrated that both serum NLR and synovial fluid NLR (SF-NLR) achieved superior diagnostic and prognostic accuracy for septic arthritis compared with conventional markers [25]. Conversely, Jiang et al. reported that neither serum NLR nor serum MLR reliably detected occult infection in patients undergoing total hip arthroplasty for septic arthritis, a discrepancy potentially explained by differences in study population, disease stage, and biomarker measurement timing [51].

Zhao et al., in a preliminary study, reported significantly elevated serum NLR and MLR in early postoperative PJIs, supporting our finding that these indices may be most informative in acute presentations [37]. From a pathophysiological standpoint, the serum NLR is a well-known and established marker for inflammation and is based on neutrophils, that have a major role in the innate immune response and are involved in the first line of defense against bacterial infections, and lymphocytes, that are involved in and orchestrate the adaptive immune response and are targeting specific pathogens [37]. It has also been reported that neutrophils reach a peak value by postoperative day 2 and preoperative values by postoperative day 21, while lymphocytes reach a low value by 2 and preoperative values by postoperative day 14 to 21 [37]. The reported lymphocytopenia is induced by various anti-inflammatory cytokines that are released in the bloodstream during infection, and can also induce immunosuppression, that is associated with apoptosis of a large number of lymphocytes, decreasing the total lymphocyte count. Elevated serum NLR values reflect a shift toward neutrophil predominance [17,18,19]; therefore, serum NLR seems to be beneficial in the diagnosis of acute PJIs [37]. Serum MLR, while conceptually appealing as a composite marker of monocyte activation and lymphocyte suppression, may lack the dynamic sensitivity of serum NLR in the PJI setting. In chronic or low-grade infections, where systemic inflammatory responses are often attenuated, both ratios may demonstrate diminished discriminative ability.

The heterogeneity of the existing literature, spanning variations in infection definition, biomarker cut-off thresholds, and patient characteristics, likely contributes to the inconsistent findings reported to date.

A notable strength of this meta-analysis lies in its comprehensive, multi-database search strategy, inclusion of contemporary studies, and stratification by infection type and case definition. The inclusion of studies across diverse geographic regions and multiple arthroplasty types enhances external validity. Involving a multinational dataset enhances generalizability of the results. However, limitations warrant careful consideration. Biomarker cut-off values varied substantially between studies, and laboratory measurement techniques were not standardized also sampling time was not taken into consideration. The predominance of retrospective study designs raises the potential for selection bias, while incomplete reporting limited the ability to perform certain subgroup analyses. Although publication bias was assessed, the possibility of selective reporting cannot be entirely excluded. The relatively small number of studies available for some subgroups, particularly EBJIS-defined infections and shoulder or elbow arthroplasties, might limit the precision and generalizability of those findings. Moderate statistical heterogeneity observed, is likely due to differences in PJI definition, chronicity, and biomarker thresholds. Some asymmetry in funnel plots suggests possible small-study effects.

From a clinical perspective, serum NLR offers a low-cost, widely accessible adjunct to the diagnostic work-up of suspected PJI, especially in acute cases where its discriminative ability appears greatest. However, our results suggest that serum NLR should not be relied upon as a standalone diagnostic tool but rather integrated with established criteria such as EBJIS, MSIS or ICM definitions and other laboratory tests including C-reactive protein (CRP), erythrocyte sedimentation rate (ESR), and synovial fluid analysis. The diagnostic role of serum MLR remains less certain; while it may have a supportive role, its performance appears inferior to that of serum NLR in most contexts.

Future research should prioritize large-scale, prospective, multicenter studies employing standardized definitions, harmonized laboratory methodologies, and consistent biomarker thresholds. Direct head-to-head comparisons of serum NLR, serum MLR, and other emerging biomarkers both individually and in combination are warranted. The development of composite biomarker panels incorporating serum NLR, serum MLR, and additional inflammatory indices, potentially integrated into predictive algorithms, may yield more accurate and clinically actionable diagnostic tools for PJI.

## 5. Conclusions

In conclusion, this meta-analysis demonstrates that serum neutrophil-to-lymphocyte ratio exhibits superior diagnostic performance compared with serum monocyte-to-lymphocyte ratio for the detection of periprosthetic joint infections, with its greatest utility observed in acute infections. Both indices are inexpensive and easily obtained from routine complete blood counts, but variability in study methodology and limited high-quality prospective data necessitate cautious interpretation. Until further validation is available, the integration of serum neutrophil-to-lymphocyte ratio and serum monocyte-to-lymphocyte ratio into multimodal diagnostic algorithms, rather than as a solitary determinant, appears to represent the most appropriate clinical application of this serum markers in the diagnosis of periprosthetic joint infections.

## Figures and Tables

**Table 1:** Data Completeness Across 29 Studies.

Variable	No. of Studies Reported	Missing
Serum NLR (PJI group)	26	3
Serum NLR (non-PJI group)	25	4
AUC (NLR)	27	2
Serum Sensitivity (serum NLR)	28	1
Serum Specificity (serum NLR)	28	1
Cut-off (serum NLR)	29	0
Serum MLR assessed in 11 articles		
Serum MLR (PJI group)	11	0
Serum MLR (non-PJI group)	11	0
AUC (serum MLR)	8	3
Sensitivity (serum MLR)	10	1
Specificity (serum MLR)	10	1
Cut-off (serum MLR)	11	0

**Table 2:** Summary of Spearman Correlations Between Diagnostic Metrics for NLR.

Variable Pair	Spearman’s ρ	*p*-Value	Strength of Correlation	Statistical Significance
AUC_NLR ↔ Sensitivity_NLR	0.562	0.003	Moderate	✔ Significant
AUC_NLR ↔ Specificity_NLR	0.461	0.018	Moderate	✔ Significant
AUC_NLR ↔ Cutoff_NLR	0.178	0.384	Weak	✘ Not Significant
Sensitivity_NLR ↔ Specificity_NLR	–0.370	0.062	Weak	✘ Not Significant
Sensitivity_NLR ↔ Cutoff_NLR	–0.218	0.285	Weak	✘ Not Significant
Specificity_NLR ↔ Cutoff_NLR	0.399	0.043	Weak to Moderate	✔ Significant

**Table 3:** Summary of Spearman Correlations Between Diagnostic Metrics for MLR.

Variable Pair	Spearman’s ρ	*p*-Value	Strength of Correlation	Statistical Significance
AUC_MLR ↔ Sensitivity_MLR	0.168	0.691	Weak	✘ Not Significant
AUC_MLR ↔ Specificity_MLR	0.571	0.139	Moderate	✘ Not Significant
AUC_MLR ↔ Cutoff_MLR	0.611	0.108	Moderate	✘ Not Significant
Sensitivity_MLR ↔ Specificity_MLR	–0.431	0.286	Weak to Moderate	✘ Not Significant
Sensitivity_MLR ↔ Cutoff_MLR	0.199	0.637	Weak	✘ Not Significant
Specificity_MLR ↔ Cutoff_MLR	0.168	0.691	Weak	✘ Not Significant

**Table 4:** HSROC pooled estimates.

Biomarker	Studies	Sensitivity	Sens_Lo	Sens_Hi	Specificity	Spec_Lo	Spec_Hi	AUC
**NLR**	25	0.703	0.653	0.748	0.709	0.660	0.755	0.755
**MLR**	8	0.690	0.608	0.762	0.724	0.619	0.809	0.750

**Table 5:** Risk of Bias Assessment for included studies.

Study Ref No.	Flow and Timing	Index Test	Patient Selection	Reference Standard
11	Moderate	Moderate	Low	Moderate
15	Moderate	Moderate	Low	Moderate
24	Moderate	Moderate	Low	Moderate
27	Moderate	Moderate	Low	Moderate
28	Low	Moderate	Low	Moderate
29	Moderate	Moderate	Low	Moderate
30	High	Moderate	Low	Moderate
31	Moderate	Moderate	Low	Moderate
32	High	Moderate	Low	Moderate
33	Moderate	Moderate	Low	Moderate
34	Moderate	Moderate	Low	Moderate
35	Moderate	Moderate	Low	Moderate
36	High	Moderate	Low	Moderate
37	Moderate	Moderate	Low	Moderate
38	Moderate	Moderate	Low	Moderate
39	Moderate	Low	Low	Moderate
40	Moderate	Unclear	Low	Moderate
41	Moderate	Moderate	Low	Moderate
42	Moderate	Moderate	Low	Moderate
43	Moderate	Moderate	Low	Moderate
44	Moderate	Moderate	Low	Moderate
45	Moderate	Moderate	Low	Moderate
46	Moderate	Moderate	Low	Moderate
47	Moderate	Moderate	Low	Moderate
48	Moderate	Moderate	Low	Moderate
49	High	High	Low	High
50	Moderate	Moderate	Low	Moderate
51	Moderate	Moderate	Low	Moderate
52	Moderate	Moderate	Low	Moderate

**Figure A1:**
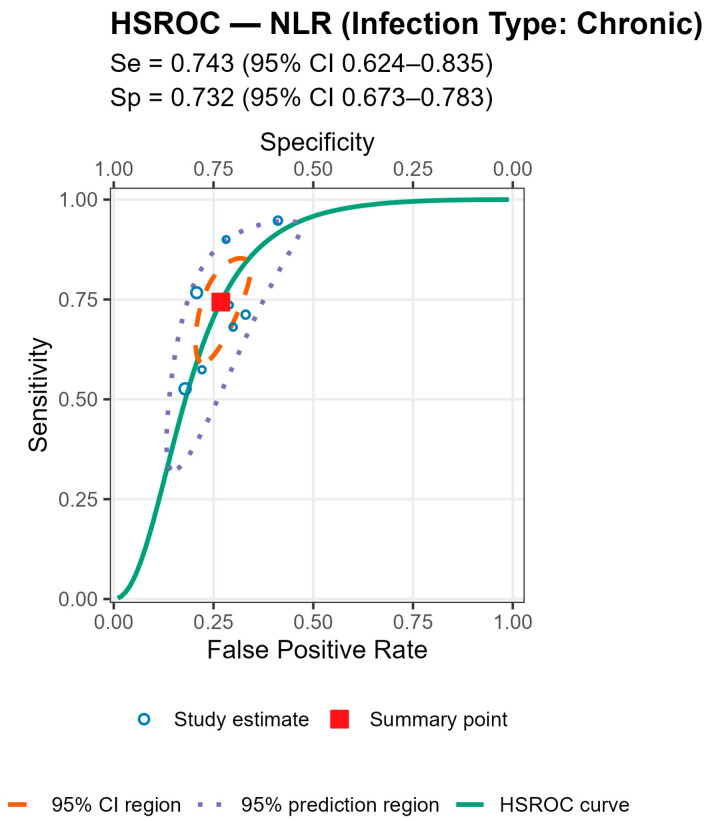
HSROC from the bivariate Reitsma model—NLR. Infection type: chronic.

**Figure A2:**
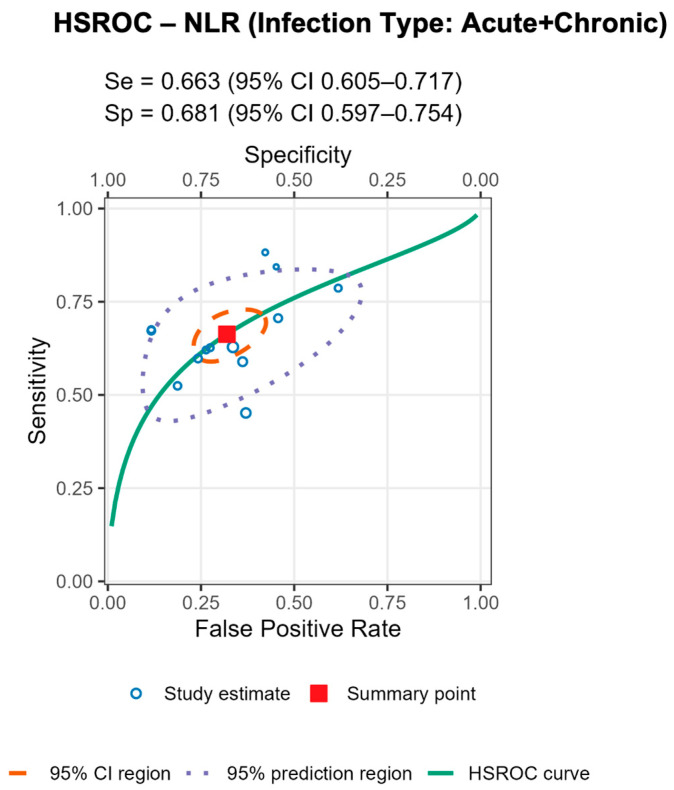
HSROC from the bivariate Reitsma model—NLR. Infection type: acute + chronic.

**Figure A3:**
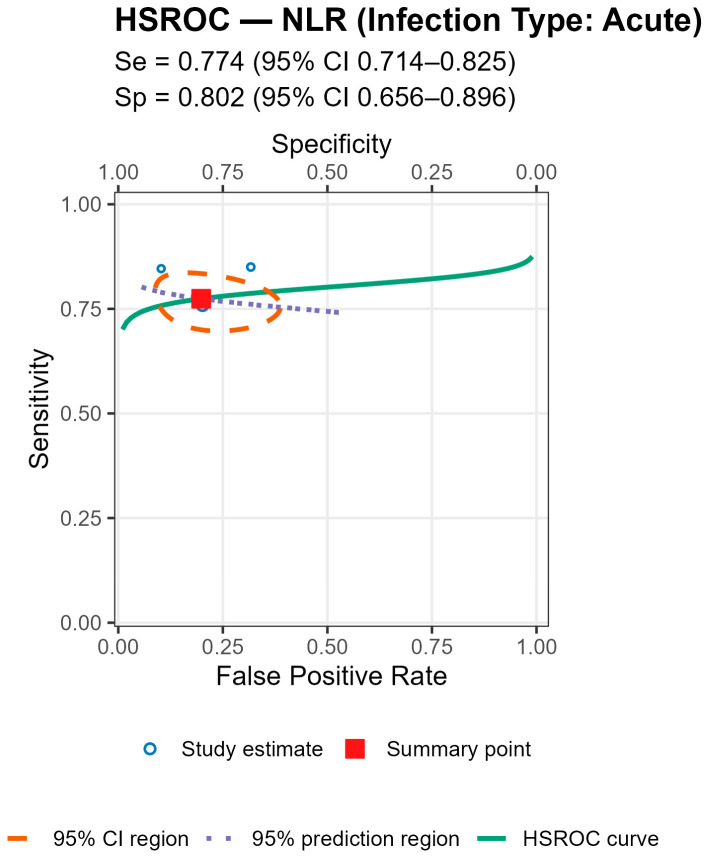
HSROC from the bivariate Reitsma model—NLR. Infection type: acute.

**Figure A4:**
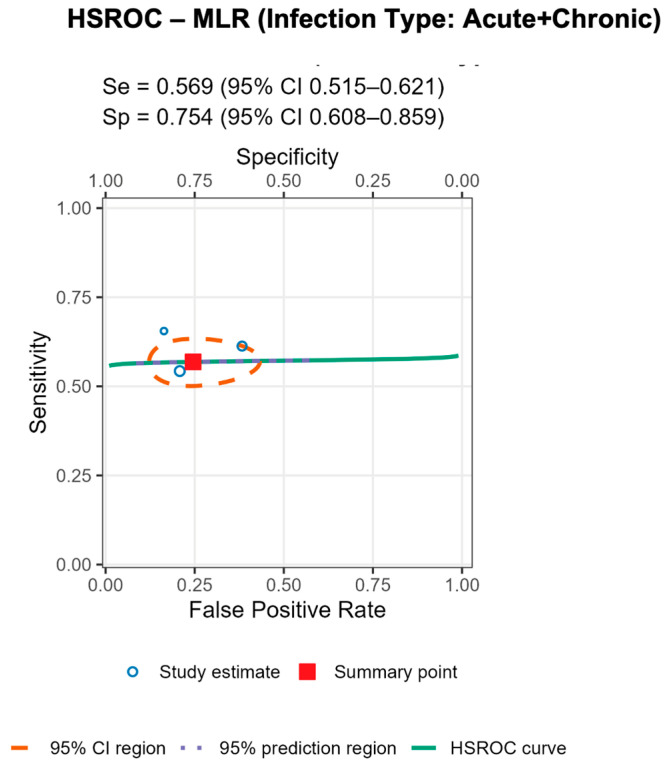
HSROC from the bivariate Reitsma model—MLR. Infection type: acute + chronic.

**Figure A5:**
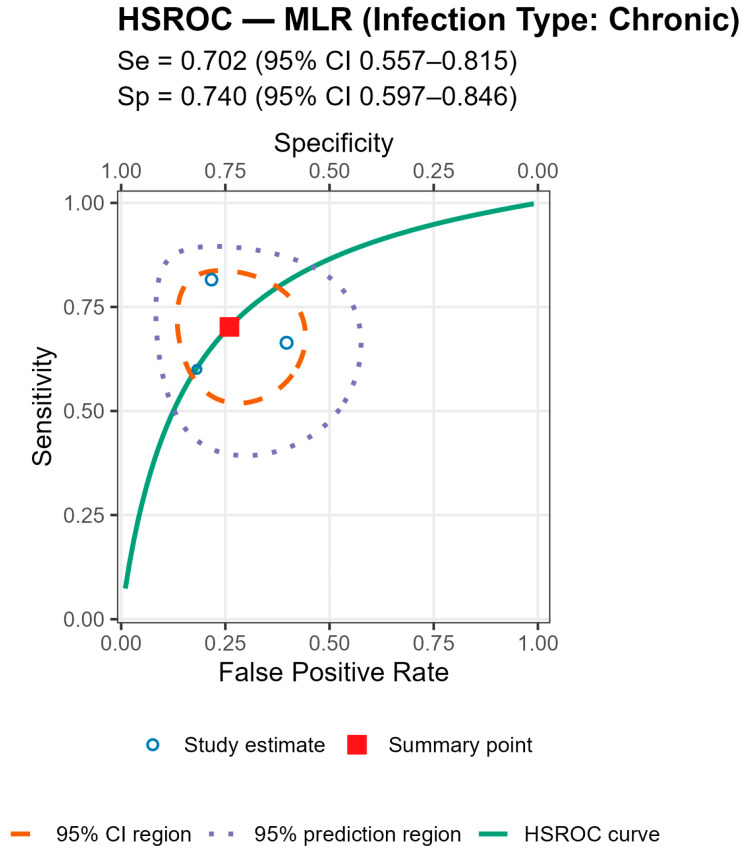
HSROC from the bivariate Reitsma model—MLR. Infection type: chronic.

**Figure A6:**
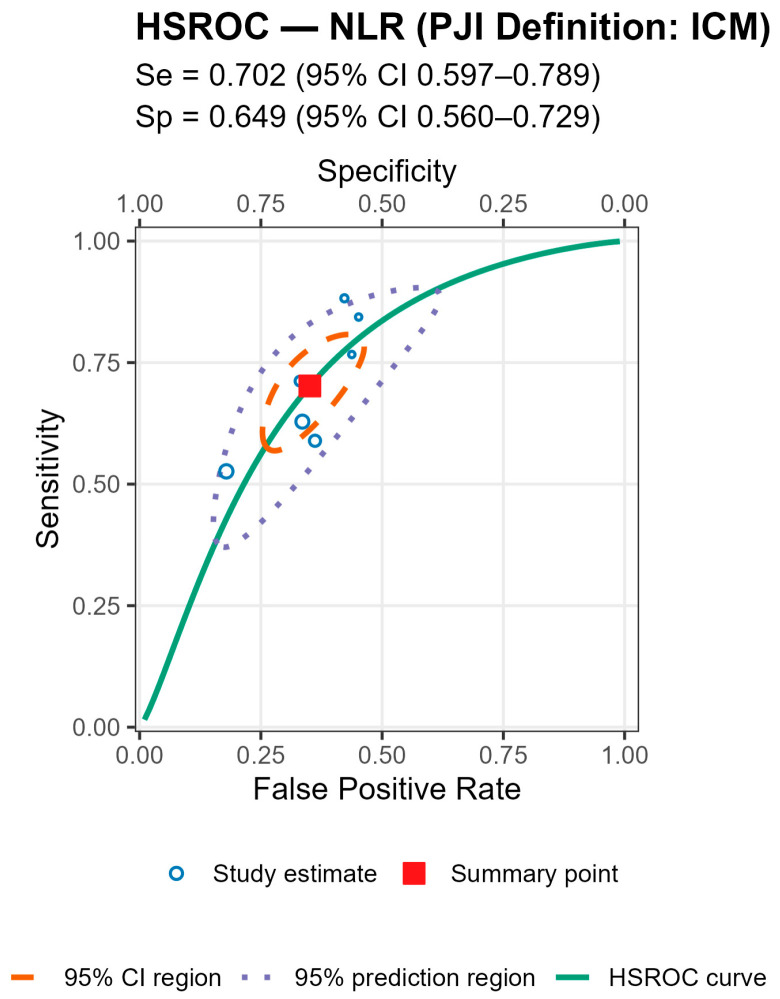
HSROC from the bivariate Reitsma model—NLR. PJI Definition: ICM.

**Figure A7:**
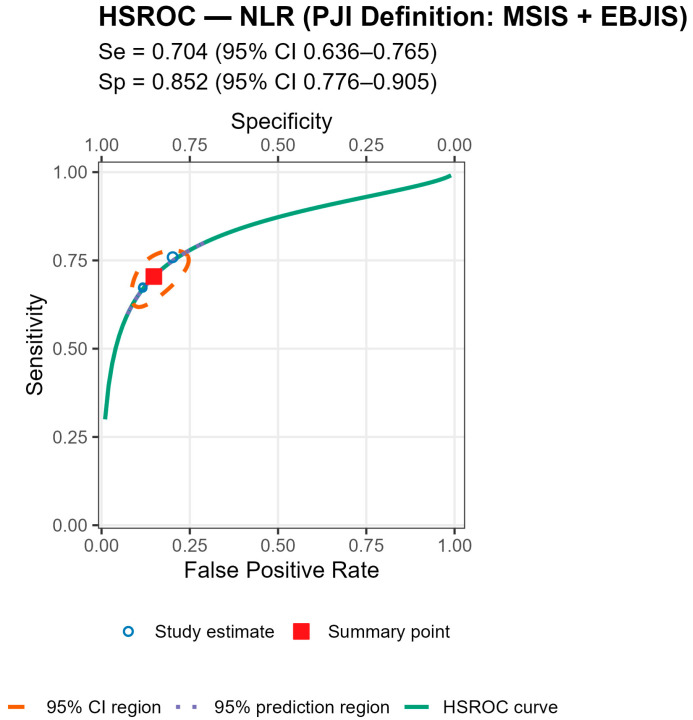
HSROC from the bivariate Reitsma model—NLR. PJI Definition: MSIS + EBJIS.

**Figure A8:**
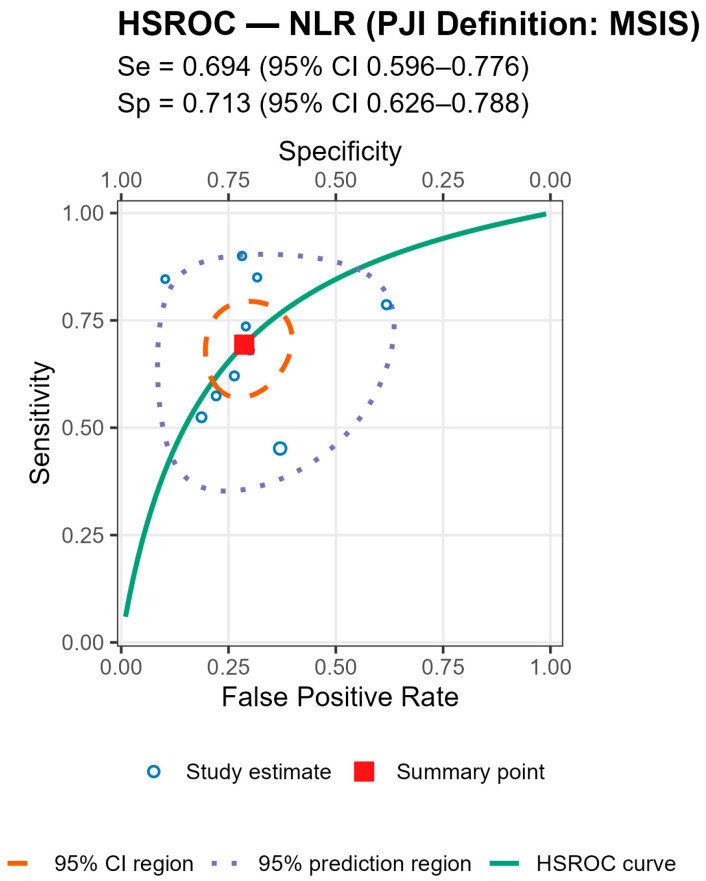
HSROC from the bivariate Reitsma model—NLR. PJI Definition: MSIS.

**Figure A9:**
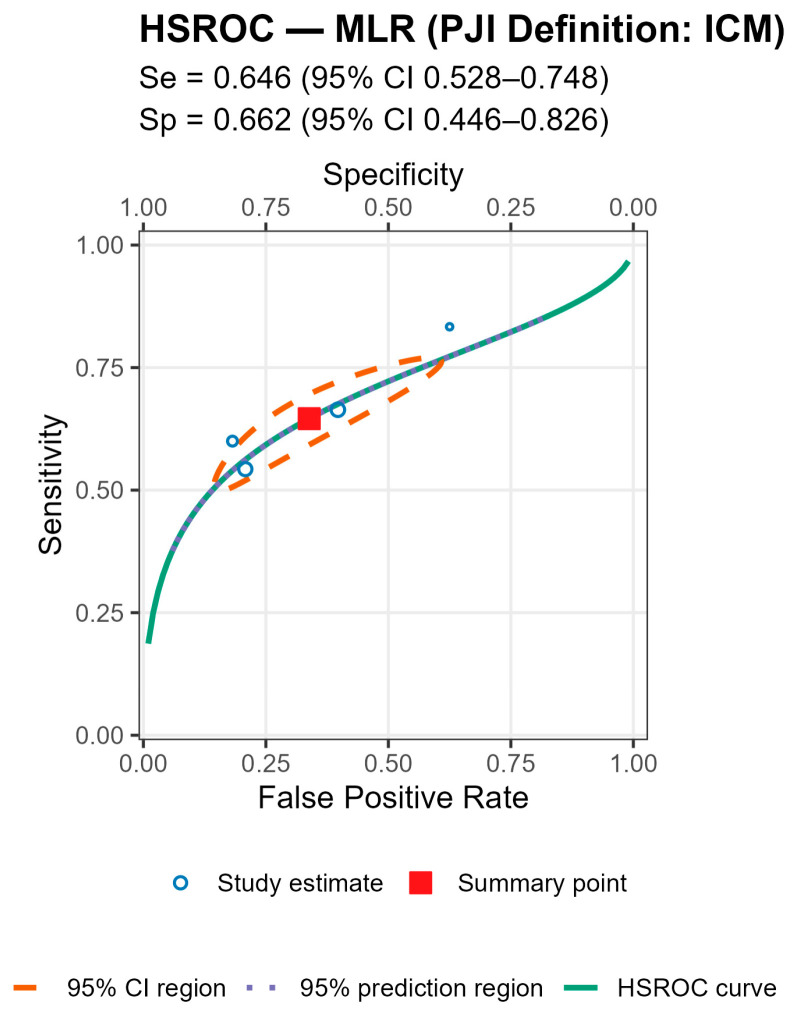
HSROC from the bivariate Reitsma model—MLR. PJI Definition: ICM.

## Data Availability

No new data were created or analyzed in this study. Data sharing is not applicable to this article.

## Supplementary Materials

The following supporting information can be downloaded at: https://www.mdpi.com/article/10.3390/jcm14217645/s1, PRISMA 2020 Abstract Checklist and PRISMA 2020 Checklist.

## Appendix A

### Appendix A.1

**Table A1:** Wald contrasts—NLR by Infection Type.

Group A	Group B	Contrast	z	*p*	Direction	Sig	Contrast Pairs
Acute	Chronic	Logit (sensitivity)	0.512	0.608	A>B	No	Acute vs. Chronic
Acute	Chronic	Logit (false positive rate)	−0.962	0.336	A<B	No	Acute vs. Chronic
Acute	Acute + Chronic	Logit (sensitivity)	2.680	0.007	A>B	Yes	Acute vs. Acute + Chronic
Acute	Acute + Chronic	Logit (false positive rate)	−1.505	0.132	A<B	No	Acute vs. Acute + Chronic
Chronic	Acute + Chronic	Logit (sensitivity)	1.245	0.213	A>B	No	Chronic vs. Acute + Chronic
Chronic	Acute + Chronic	Logit (false positive rate)	−1.056	0.291	A<B	No	Chronic vs. Acute + Chronic

Wald contrasts between subgroups (logit scale). “A > B”—Group A has a higher value of the reported logit metric than Group B; for logit(sensitivity) this implies higher pooled sensitivity, while for logit(false positive rate) it implies a higher FPR (i.e., lower specificity). Two-sided Wald test; significance at *p* < 0.05. Values < 0.001 are reported as “<0.001”.

Only one contrast reached significance: Acute vs. Acute + Chronic for logit (sensitivity). Acute shows higher pooled sensitivity than mixed acute + chronic studies. No significant differences in false-positive rate (specificity proxy) were seen.

### Appendix A.2

**Table A2:** Wald contrasts—MLR by Infection Type.

Group A	Group B	Contrast	z	*p*	Direction	Sig	Contrast Pairs
Chronic	Acute + Chronic	logit (sensitivity)	1.717	0.086	A>B	No	Chronic vs. Acute + Chronic
Chronic	Acute + Chronic	logit (false positive rate)	0.156	0.876	A>B	No	Chronic vs. Acute + Chronic

Wald contrasts between subgroups (logit scale). “A > B”—Group A has a higher value of the reported logit metric than Group B; for logit(sensitivity) this implies higher pooled sensitivity, while for logit(false positive rate) it implies a higher FPR (i.e., lower specificity). Two-sided Wald test; significance at *p* < 0.05. Values < 0.001 are reported as “<0.001”.

No significant subgroup differences by infection type were observed (i.e., a weak trend toward higher sensitivity in Chronic vs. Acute+Chronic did not reach p<0.05).

### Appendix A.3

**Table A3:** Wald contrasts—NLR by PJI Definition.

Group A	Group B	Contrast	z	*p*	Direction	Sig	Contrast Pairs
MSIS	ICM	Logit (sensitivity)	−0.124	0.901	A<B	No	MSIS vs. ICM
MSIS	ICM	Logit (false positive rate)	−1.067	0.286	A<B	No	MSIS vs. ICM
MSIS	MSIS + EBJIS	Logit (sensitivity)	−0.191	0.848	A<B	No	MSIS vs. MSIS + EBJIS
MSIS	MSIS + EBJIS	Logit (false positive rate)	2.542	0.011	A>B	Yes	MSIS vs. MSIS + EBJIS
ICM	MSIS + EBJIS	Logit (sensitivity)	−0.040	0.968	A<B	No	ICM vs. MSIS + EBJIS
ICM	MSIS + EBJIS	Logit (false positive rate)	3.529	<0.001	A>B	Yes	ICM vs. MSIS + EBJIS

Wald contrasts between subgroups (logit scale). “A > B”—Group A has a higher value of the reported logit metric than Group B; for logit(sensitivity) this implies higher pooled sensitivity, while for logit(false positive rate) it implies a higher FPR (i.e., lower specificity). Two-sided Wald test; significance at *p* < 0.05. Values < 0.001 are reported as “<0.001”.

Sensitivity did not differ by PJI definition. However, MSIS and ICM each showed higher false-positive rates than MSIS+EBJIS, implying worse specificity under MSIS or ICM vs. the combined MSIS + EBJIS definition.

#### MLR by PJI Definition

No contrasts available, due to insufficient studies per subgroup to fit the model and compute variances.
